# Multi-Organ Toxicity of Combined PFOS/PS Exposure and Its Application in Network Toxicology

**DOI:** 10.3390/biology14121714

**Published:** 2025-11-30

**Authors:** Qi Liu, Xianghui Ma, Jiaming Liu, Yan Liu

**Affiliations:** College of Life and Science and Technology, Harbin Normal University, Harbin 150025, China; liuqi@hrbnu.edu.cn (Q.L.); maxianghui2001@163.com (X.M.); 15661695438@163.com (J.L.)

**Keywords:** PFOS, PS, inflammation, oxidative stress, network toxicology, molecular docking

## Abstract

Perfluorooctane sulfonate (PFOS) has been widely utilized in products such as cotton textiles, hydraulic oils, coatings, pharmaceuticals, cosmetics, etc. Now it is widely distributed in various environmental media, wildlife, and human bodies. Polystyrene (PS) as a kind of plastics, their products under the physical, chemical, and biological decomposition in the environment are widely distributed in the air, soil, oceans, surface water, and sediments. However, PS and PFOS often coexist in the environment, making the study of their combined exposure mechanisms more aligned with actual conditions. This research integrates network toxicology and molecular biology techniques to predict the toxicity and common differentially expressed gene enrichment pathways of PFOS and PS. This study investigates the toxic effects of combined exposure to PFOS and PS on the mouse growth and development, immune functions, and other aspects. Additionally, it delves into the expression differences in various genes in mice after stimulation by PFOS and PS, the pathological changes in multiple organs, and the toxic effects on organs such as the liver, kidneys, and intestines. The results reveal that combined exposure to PFOS and PS does not significantly damage the kidney but leads to morphological damage in the liver and intestinal tissues, reduced antioxidant capacity, and the occurrence of inflammation. Based on the network toxicology findings, it is hypothesized that during combined exposure to PFOS and PS, the exacerbation of inflammatory responses further mediates the reduction in antioxidant capacity and the intensification of oxidative stress, ultimately resulting in tissue damage. This study provides innovative theoretical and research directions for the detection and prevention of combined exposure to PFOS and PS, offering a new paradigm for toxicological research, with significant theoretical and practical implications.

## Simple Summary

Perfluorooctane sulfonate (PFOS) and polystyrene (PS) microplastics pose significant threats to environmental health due to their persistence and bioaccumulation. Co-exposure to these substances may induce synergistic multi-organ toxicity through complex mechanisms, necessitating advanced toxicological methods for risk assessment. This study employed network toxicology techniques through the following steps: Multi-organ toxicity study: Demonstrating that the co-exposure of PFOS and PS can cause varying degrees of liver, kidney, and intestinal damage through reduced antioxidant capacity. Target identification: Extracting potential functional targets of PFOS and PS from the CTD and GeneCards databases, followed by Venn analysis to identify overlapping targets. Protein interaction network analysis: Identifying hub genes (e.g., *TNF*, *TP53*, etc.) using STRING database and Cytoscape (Version 3.9.1) software. Molecular docking: Verifying the binding energy and interaction sites of PFOS/PS with liver damage-associated proteins (e.g., *TNF*, *IL6*, *IL1B*) through AutoDock Vina (Version 1.1.2) and PyMOL (Version 3.1). Conclusion: Network toxicology reveals novel pathways (e.g., inflammatory–cell death crosstalk) in PFOS/PS co-exposure, providing predictive tools for risk stratification.

## 1. Introduction

Over the past few decades, due to rapid industrial development, the types and production volumes of chemicals worldwide have surged dramatically. Some intermediate products and metabolic wastes, released untreated into natural environments, have caused severe harm to ecosystems and human health. Four categories of emerging contaminants of international concern are persistent organic pollutants (POPs) [1], endocrine-disrupting compounds (EDCs) [2], antibiotics [3], and microplastics [4]. These substances, once discharged into the environment, will become emerging contaminants. Most emerging contaminants exhibit carcinogenic, teratogenic, and mutagenic effects on organisms, disrupt endocrine systems, and thereby threaten human survival and reproduction. Representative POPs that are widely used include pentachlorophenol, perfluorooctane sulfonate compounds, and polytetrafluoroethylene nanoparticles [5].

Since industrialized production began in the 1950s, PFOS has been extensively applied in products such as cotton fabrics, mechanical lubricants, hydraulic oils, coatings, refrigerants, surfactants, paper surface treatments, firefighting foams, pesticides, pharmaceuticals, and cosmetics [6]. Studies show that PFOS resists degradation even after prolonged exposure to strong oxidizers or boiling in acidic solutions, demonstrating water solubility and a widespread presence in environmental media, wildlife, and human tissues. Toxicological studies reveal high absorption rates of PFOS in animals, bioaccumulation along food chains, and adverse effects including hepatoxicity [7], kidney injury [8], male reproductive toxicity [9], neurotoxicity [10], mitochondrial energy metabolism disorders, lipid metabolism disturbances, and oxidative stress induction [11]. Despite strict usage restrictions in China, PFOS remains detectable in tap water, air, and certain foods. Survey data indicate urban tap water PFOS concentrations up to 100 ng/L, with human exposure primarily through contaminated water and food. Due to its ubiquity, persistence, and bioaccumulation, PFOS is detectable in over 99% of the general population at blood levels of 20–30 ng/mL [12]. In 2023, China’s Ministry of Ecology and Environment included PFOS in its list of key controlled emerging pollutants, prohibiting its production and use (except for firefighting foam agents). In environmental settings, PFOS is primarily released as solids into soil and water bodies, with detected concentrations as low as 0.59 ng/L in rainwater samples from Canadian cities [13]. A study across Chinese urban water samples found PFOS in all water types (surface water, seawater, groundwater, municipal water, industrial wastewater, and tap water), indicating widespread contamination at approximately 1 ng/L levels, potentially causing irreversible damage to humans and animals through long-term consumption [14].

Plastics, hailed as one of the greatest inventions of the 20th century, have been widely adopted across industries due to their superior properties. However, their extensive use has led to severe environmental issues. Statistical research indicated that between 1950 and 2015, approximately 4.9 billion tons of plastic waste accumulated in landfills or were released into natural environments [15,16]. According to Plastics Europe’s latest report, global plastic production exceeded 400 million tons in 2022, with China accounting for 32% of the total. Projections indicate that discarded plastic waste in natural environments could reach 120 billion tons by 2050 [17]. These plastics undergo physical, chemical, and biological fragmentation in natural conditions, forming macroplastics, mesoplastics, microplastics (PSs, <5 mm), and nanoplastics (NPs, <100 nm), posing severe ecological threats. Primary sources include firefighting foams, textile waterproof agents, mechanical abrasion, UV degradation, hydrolysis, biodegradation, automobile tires, and agricultural plastic films. Common environmental microplastics comprise polyethylene (PE), polyvinyl chloride (PVC), polystyrene (PS), polyamide (PA), and polyethylene terephthalate (PET), which are widely distributed in air, soil, oceans, surface waters, and sediments [18]. Inhaled airborne microplastic particles deposit in lungs, causing female reproductive toxicity and pyroptosis and inflammation in mouse kidneys [19,20,21]. In recent years, microplastics released from disposable plastic tableware, bags, and food containers under high-temperature conditions have posed significant health risks, entering human bodies directly through drinking water, seafood (73% of deep-sea fish tested positive for microplastics), and takeout containers. Among these, PS exhibits multi-medium, multi-scale distribution characteristics in environments, with pollution levels closely related to environmental media, human activity intensity, and plastic morphology. In recent years, the Pacific garbage patch has formed high-concentration aggregation zones, with over 5 trillion plastic particles floating on surface waters, where PS constitutes a significant proportion. These particles can breach biological barriers, enter human bodies through food chains, and pose severe health risks, with studies detecting PS in 36% of human blood samples. PFOS and PS in nature ultimately harm human health through drinking water, illegal additives in daily products, and food chain bioaccumulation. In actual environments, PS often coexists with PFOS, raising critical questions about whether PS can adsorb PFOS as a carrier, exacerbating toxicity, and whether combined exposure causes more severe toxic responses and organ damage than individual exposure.

Network toxicology is an interdisciplinary approach integrating bioinformatics, systems biology, and chemoinformatics to predict potential targets and the enrichment of toxicity through specific modeling, systematically analyzing the toxicological characteristics of compounds [22,23]. Compared with traditional toxicology technology, network toxicology focuses more on whole-system effects and interconnected toxic pathways but lacks comprehensive validation through systematic animal studies. Therefore, this study combines network toxicology, bioinformatics analysis, and molecular biology techniques to predict toxicity and differentially expressed gene enrichment pathways of PFOS and PS. Using internationally standardized laboratory mice as subjects and in vitro models, we investigate the toxic effects of combined PFOS and PS exposure on mouse growth, development, and immune functions. By integrating network toxicology predictions, we explore synergistic relationships during combined exposure, identify targets and signaling pathways for multi-organ damage, and provide innovative theoretical directions for the detection and prevention of PFOS/PS exposure in the environment. This research establishes a new paradigm for toxicological studies, offering significant theoretical and practical implications.

## 2. Materials and Methods

All experimental procedures on animals in this study were conducted in accordance with the laboratory animals care guidelines and approved by the Institutional Animal Care and Use Committee of Harbin Normal University.

### 2.1. Animals and Treatment

Healthy male *Kunming mice*, aged 8 weeks, were obtained from Liaoning Changsheng Biotechnology Co., Ltd. (Benxi, China). The mice were acclimated to standard laboratory conditions, including a 12 h light/dark cycle, a room temperature of 20–22 °C, and a relative humidity of 50–60%, for 1 week prior to the start of the experiments. All experiments were approved by the Animal Ethics Committee of Harbin Normal University, China.

PFOS (CDAA-S-3110032-AD-1.2 mL, Shanghai, China) was purchased from Anpel Laboratory Technologies (Shanghai) Inc. (Shanghai, China), PS (CFEQ-4-550050-0100, Shanghai, China) was purchased from (6-1-0500, Tianjin, China, Tianjin Beisile Chromatography Technology Development Center). Prior to the experiment, the PS was ultrasonically processed for 30 min. The PS MPs suspension used in the experiment was prepared by diluting the purchased PS MPs stock solution with distilled water to a concentration of 5 mg/L, followed by ultrasonication to ensure homogeneity in the distilled water. The PFOS was directly diluted with ultrapure water to achieve a final application concentration of 5 mg/kg. The selected dose accounts for the bioaccumulative properties and trophic magnification potential of PFOS + PS, while ensuring observable biological responses within a limited experimental time frame.

Thirty male *Kunming mice* aged 8 weeks (20–25 g) were randomly assigned to three groups: NC (Control), PFOS, and PFOS + PS. Ten mice per group ensured sufficient statistical power. The animals received 12 h light–dark cycles daily with free access to food and water. Pre-experiment concentration screening identified optimal exposure levels for PFOS (5 mg/kg) and PS (5 mg/L) through dose–response curves. Control groups included solvent-only drinking water. The solution was replaced every 24 h to maintain consistent concentrations, with all groups maintained for 8 weeks. All the animals consumed food and drinking water freely for the whole study. After the successful establishment of the model, the blood and liver tissue of the mice were collected for biochemical, histopathological examination and subsequent experiments. The selected dose accounts for the bioaccumulative properties and trophic magnification potential of PFOS + PS, while ensuring observable biological responses within a limited experimental time frame.

### 2.2. Histology Observation of Tissues

The fresh collected tissue including liver, kidney, and colon was fixed with fresh tissue fixative solution for more than 24 h. The instructions for this stage were as follows: Remove the tissue and trim the target tissue with a scalpel in the ventilation cupboard, then put the trimmed tissue and a label in the dehydration box. Dehydration and wax leaching: put the dehydration box into the dehydrator in order to dehydrate with an alcohol gradient, with 75% alcohol 4 h, 85% alcohol 2 h, 90% alcohol 2 h, 95% alcohol 1 h, anhydrous ethanol I 30 min, anhydrous ethanol II 30 min, alcohol benzene 5–10 min, xylene II 5–10 min, 65 °C melting paraffin I 1 h, 65 °C melting paraffin II 1 h, and 65 °C melting paraffin III 1 h. Embed wax-soaked tissue in the embedding machine. First, put the melted wax into the embedding frame, and before the wax solidifies, remove the tissue from the dewatering box and put it into the embedding frame according to the requirements of the embedding surface and affix the corresponding label. Cool down at a −20 °C freezing table. After the wax is solidified, remove it from the embedded frame and repair. Place the trimmed wax block at a −20 °C freezing table, then slice the modified tissue chip wax block on the 4 μm paraffin slicer. The tissue was flattened when the slice floats on the 40 °C water of the spreading machine, and the tissue was picked up by the glass slides and baked in the oven at 60 °C. After the water-baked dried wax has melted, it is taken out and stored at room temperature. After preparing the sections, they were stained with hematoxylin and eosin (H&E) and observed under a microscope.

### 2.3. Antioxidant Enzyme Activities and Content

Free radical scavenging enzymes such as total antioxidant capacity (T-AOC), Catalase (CAT), and malondialdehyde (MDA) as an index of oxidative damage were determined to compare the differences between NC, PFOS, PFOS + PS group liver, kidney, and colon. Commercial assay kits for T-AOC, CAT, and MDA were provided by Jiancheng Biotechnology Research Institute (Nanjing, China).

### 2.4. Toxicity Prediction Analysis

ADMETlab 3.0 (https://admetlab3.scbdd.com/, accessed on 26 November 2025) is a database used for the systematic assessment of a compound’s absorption, distribution, metabolism, excretion, and toxicity. These structures and information were then input into ADMETlab 3.0 for the toxicity prediction and analysis of PFOS and PS.

### 2.5. Acquisition of PFOS/PS and Liver Injury-Related Target Genes

The SMILES sequence of PS (D011137, C(=C)C1=CC=CC=C1) and PFOS (C076994, C(C(C(C(C(F)(F)S(=O)(=O)O)(F)F)(F)F)(F)F)(C(C(C(F)(F)F)(F)F)(F)F)(F)F) was obtained from the PubChem database. To identify PFOS + PS exposure related target genes, data were retrieved from the Comparative Toxicogenomics Database (CTD), Swiss Target Prediction, and STITCH, with duplicate entries removed. For liver injury-related target genes, the GeneCards and NCBI databases were queried using “liver injury” as the keyword, and duplicate genes were eliminated.

### 2.6. Construction of the Protein–Protein Interaction

Network and Enrichment Analysis: The PFOS + PS exposure related genes and liver injury-associated genes were subjected to Venn analysis to identify intersected target genes. These intersected target genes were uploaded to the STRING database (https://cn.string-db.org/) (organisms set to *Mus musculus*) to construct a Protein–Protein Interaction (PPI) network. A minimum required interaction score of 0.9 was applied, and unconnected genes were removed to improve network specificity. The resulting PPI network was exported for further analysis. Subsequently, the genes identified from the PPI network were subjected to Gene Ontology (GO) and Kyoto Encyclopedia of Genes and Genomes (KEGG) pathway enrichment analysis using the DAVID database, with the organisms set to *Mus musculus*.

### 2.7. Core Target Screening

The CentiScaPe 2.2 plugin in Cytoscape (version 3.9.1) was used to analyze the degree centrality, betweenness centrality, and closeness centrality of each gene within the PPI network. The median values of these centrality measures were calculated separately. Genes with values exceeding the median for all three centralities were identified as core targets involved in PFOS + PS exposure-induced liver injury.

### 2.8. Molecular Docking

To further investigate the interaction between PFOS + PS and core target proteins, molecular docking software was used to predict the binding mode and binding energy of PFOS + PS with its targets. The 3D structures of PFOS + PS and target proteins were obtained from PubChem and UniProt (organisms set to *Mus musculus*), respectively. PyMOL software (version 3.1) was used to preprocess the molecules by removing water, adding hydrogen atoms, and calculating charges. AutoDock Tools software (version 1.5.7) prepared the receptor and ligand files, and the docking box was set to define the binding site. Docking was performed using AutoDock Vina, and PyMOL was used to visualize the results, analyzing the binding mode and binding affinity between PFOS + PS and its core targets.

### 2.9. Integrated Network Toxicology

To identify the key pathways involved in PFOS + PS-induced hepatotoxicity, a comparative analysis was conducted between the top 20 enriched pathways from network toxicology analysis and the top 20 enriched pathways from transcriptomic analysis. Overlapping pathways were considered key toxicological pathways and were prioritized for further investigation. Then, a target–pathway interaction network was constructed to visualize the relationships between core targets and key pathways. Spearman’s correlation analysis was performed on DEGs and differentially abundant metabolites from transcriptomic and metabolomic data sets. Based on the correlation analysis results, data with a *p* < 0.05 were selected to construct a correlation chord diagram.

### 2.10. Statistical Analysis

Data shown in graphs are mean ± SD. All values are calculated from at least three independent biological replicates, unless specifically stated. In all tests (except survival curves), the two-tailed unpaired *t*-test was used; survival curves were conducted under log-rank test and a 95% confidence interval was used. Statistical analysis was performed using GraphPad Prism 8, SPSS 19 and Microsoft Excel 2017.

## 3. Results

### 3.1. Histomorphological Results of Organs During Exposure to PFOS and PS

**Figure 1:**
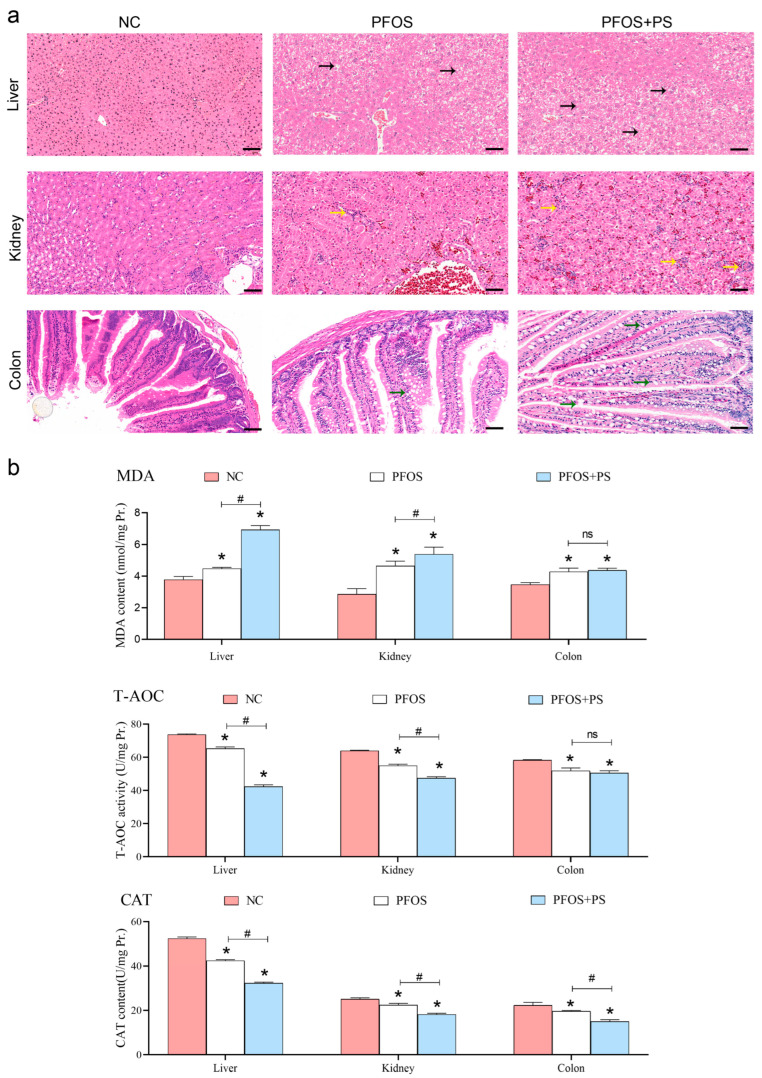
Histomorphological observation and antioxidant enzyme detection. (**a**): Microstructure of liver, kidney, and colon from different treatment groups during exposure (×200), scale bar 50 μm; (**b**): antioxidant enzyme activity and content detection. * indicates significant differences compared to the control group (*p* < 0.05); # indicates significant differences between PFOS and PFOS + PS groups (*p* < 0.05); ns indicates no significant differences (*p* > 0.05).

In Figure 1a, showing the PFOS and PS exposure groups, a significant number of hepatocytes exhibited swelling with prominent ballooning degeneration, accompanied by the disappearance of nucleoli in some liver cells (black arrows). In renal tissues, glomeruli and renal tubules showed alterations in size and number. The PFOS group displayed cellular swelling, while the combined PFOS and PS exposure group exhibited swelling, cytoplasmic loosening, vacuolar degeneration in some cells, as well as nuclear condensation, fragmentation, or dissolution hallmarks of cellular necrosis. The infiltration of inflammatory cells was observed in some areas (yellow arrows). Additionally, goblet cell numbers decreased in the intestines, with intestinal gland hyperplasia and the disruption of normal intestinal architecture. The intestinal villus epithelial cells contain a large number of vacuoles, and some cells are swollen due to the vacuoles, which will affect the intestinal function (green arrows). These findings suggest that combined exposure to PFOS and PS induces varying degrees of morphological damage in the liver, kidneys, and intestines. However, the liver injury was the most significant, which was consistent with the previous network toxicology results.

### 3.2. Results of Antioxidant Enzyme Activity and Content in Tissues

As shown in Figure 1b, tissue homogenates from all groups were analyzed for antioxidant parameters. The results showed that T-AOC and CAT activity in the liver and kidney were significantly lower (*p* < 0.05) in the PFOS/PS co-exposure group compared with the PFOS-only exposure and control groups, while MDA levels were significantly higher (*p* < 0.05). In intestinal tissues, the MDA content in both the PFOS-exposed group and the PFOS + PS-exposed group was significantly increased compared with the control group (*p* < 0.05), but no significant difference was observed between the PFOS + PS group and the PFOS-alone group (*p* > 0.05). Compared with the control group, T-AOC activity significantly decreased after exposure to PFOS or PS (*p* < 0.05), with no significant difference between the PFOS group and the PFOS + PS group (*p* > 0.05). CAT levels were reduced in both PFOS and PS exposure groups; however, intriguingly, the difference between the PFOS + PS group and the control group was not statistically significant (*p* > 0.05).

### 3.3. Physical/Chemical Properties and Toxicity Prediction of PFOS and PS

**Figure 2:**
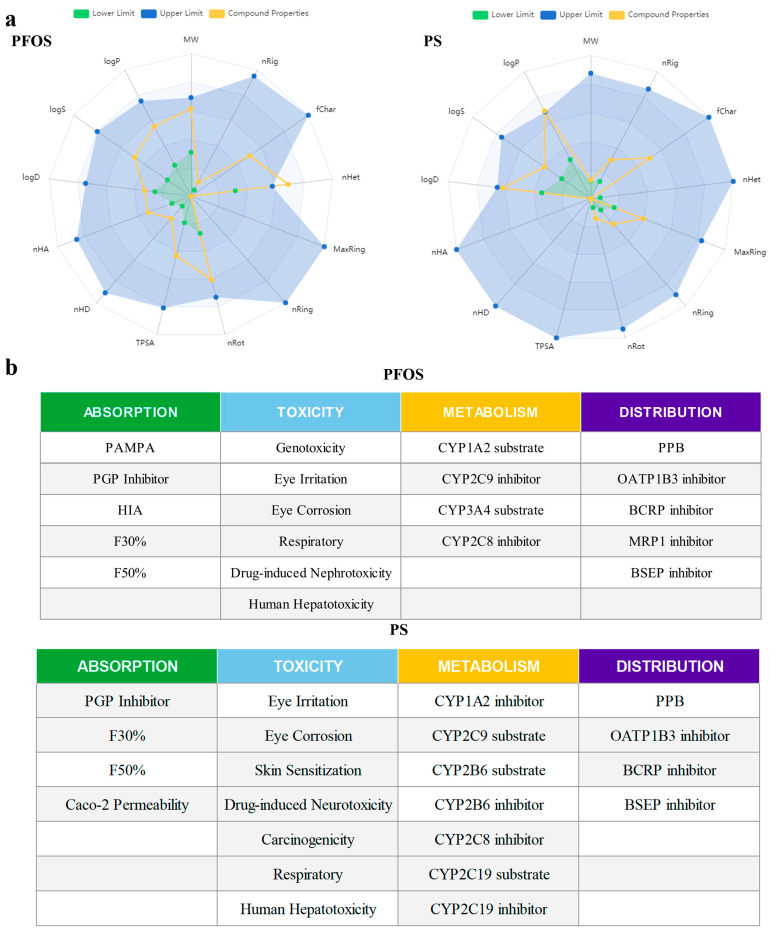
Physical/chemical properties prediction of PFOS and PS. (**a**): Limits and compound properties prediction of PFOS and PS; (**b**): physical/chemical properties prediction of PFOS/PS.

As shown in Figure 2, the physicochemical properties and performance of PFOS and PS were evaluated across multiple dimensions (absorption, distribution, metabolism, and toxicity) using ADMETlab 3.0. The results in Figure 2 indicate that PFOS has a high probability of involvement in *PAMPA*, *Pgp* inhibition, and human intestinal absorption (HIA). In terms of metabolism, PFOS may participate in the regulation of *CYP1A2* and *CYP3A4* substrates, as well as the metabolism of *CYP2C9* and *CYP2C8* inhibitors. When exposed alone, its predicted toxicity manifests in various forms, including genotoxicity, respiratory toxicity, drug-induced nephrotoxicity, human hepatotoxicity, ototoxicity, and hematotoxicity. Regarding PS absorption, it plays a key role in *Pgp* inhibition and *Caco-2* permeability. In terms of metabolism, PS is closely associated with *CYP1A2*, *CYP2C19*, *CYP2B6*, and *CYP2C8* inhibitors. When exposed alone, the predicted toxicity of PS includes drug-induced neurotoxicity, carcinogenicity, respiratory effects, human hepatotoxicity, and ototoxicity. In terms of distribution, PFOS is primarily found in the forms of *PPB*, *OATP1B3* inhibitors, *BCRP* inhibitors, *MRP1* inhibitors, and *BSEP* inhibitors, while PS is distributed in the forms of *PPB*, *OATP1B3* inhibitors, *BCRP* inhibitors, and *BSEP* inhibitors. Based on these findings, combined with previous histopathological observations of the liver, kidneys, and intestines, we conducted a comprehensive analysis and inferred that the combined exposure to PFOS and PS may significantly exacerbate liver damage induced by individual exposure, likely through *CYP* enzyme system dysfunction and metabolic disruption.

### 3.4. Target Prediction of PFOS and PS

**Figure 3:**
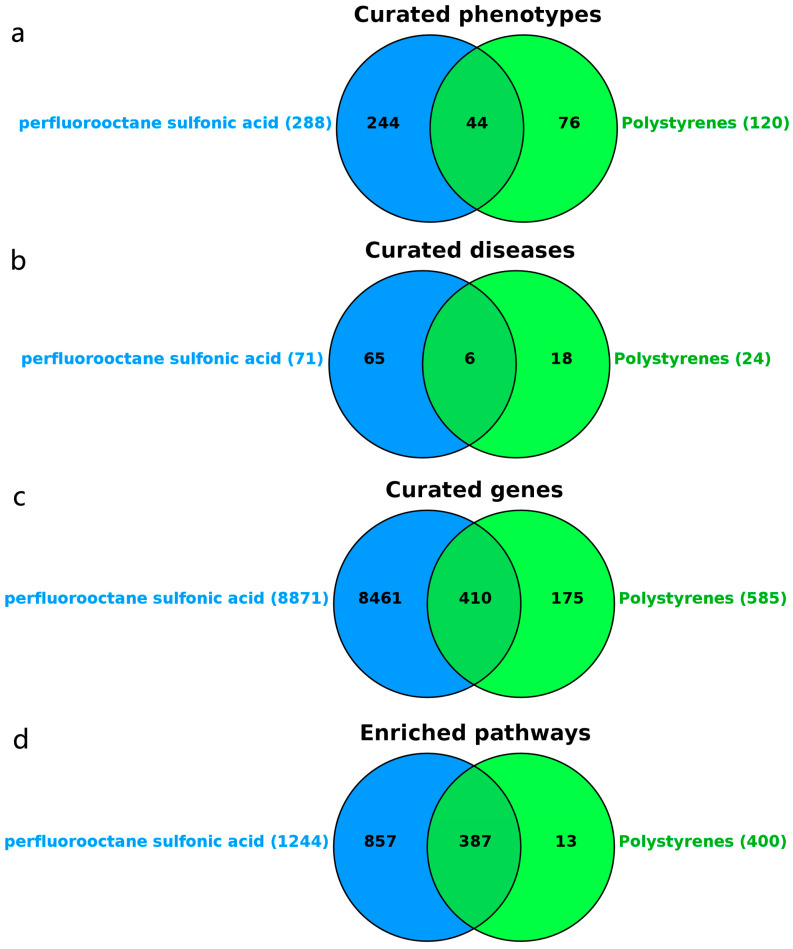
Target prediction of PFOS and PS via CTD database. (**a**): Curated phenotypes during PFOS and PS exposure; (**b**): curated diseases during PFOS and PS exposure; (**c**): curated genes during PFOS and PS exposure; (**d**): enriched pathways during PFOS and PS exposure.

In Figure 3, the CTD prediction results of PFOS and PS include curated genes, curated phenotypes, curated diseases, and enriched pathways. Figure 3a, showing the analytical results, reveals 288 phenotypes associated with PFOS and 120 phenotypes associated with PS, with 44 overlapping phenotypes including antioxidant activity, apoptosis, inflammatory response, lipid metabolism, the positive regulation of cell death, and ROS metabolism. Additionally, in Figure 3b, 71 diseases were linked to PFOS and 24 to PS, with 6 overlapping diseases: chemical and drug-induced liver injury, immune system diseases, oligospermia, testicular diseases, weight gain, and weight loss. In Figure 3c, the analysis identified 8871 genes associated with PFOS and 585 with PS, with 410 overlapping genes including key factors such as *APOE*, *ATG5*, *ATG12*, *BAX*, *BCL2*, *CASP1*, *CASP8*, *CASP9*, *CAT*, *FOXO3*, *FTH1*, *GPX4*, *GSDMD*, *IL1B*, and *IL6*, which are involved in cell death and lipid metabolism. Furthermore, as shown in Figure 3d, 1244 signaling pathways were associated with PFOS and 400 with PS, with 387 overlapping pathways including apoptosis, autophagy, ferroptosis, the *FOXO* signaling pathway, the *IL17* signaling pathway, and the *P53* signaling pathway. These findings suggest that exposure to PFOS and PS may significantly regulate processes such as inflammation, oxidative stress, and cell death.

### 3.5. Prediction of Liver Injury Disease Targets

**Figure 4:**
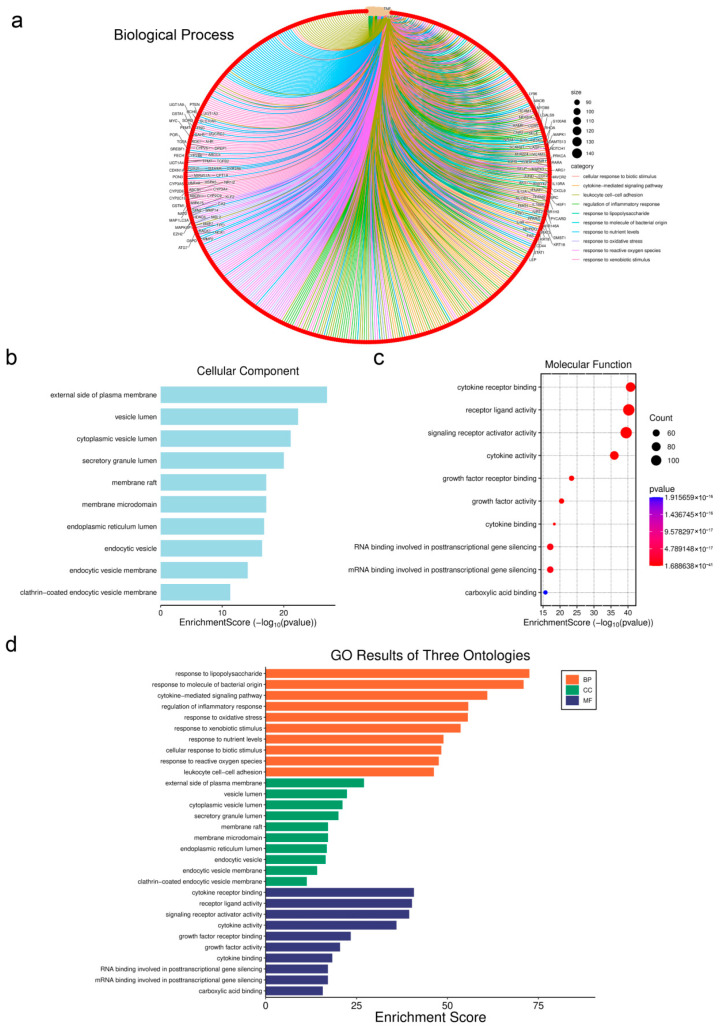
KEGG and GO enrichment analysis of liver injury. (**a**): KEGG enrichment of biological process during PFOS and PS exposure; (**b**): KEGG enrichment of cellular component during PFOS and PS exposure; (**c**): KEGG enrichment of molecular function during PFOS and PS exposure; (**d**): GO enrichment results of three ontologies.

As shown in Figure 4, we conducted separate searches using the Gencards and OMIM databases for liver injury-related targets; the results were collected using the Wei Sheng Xin online website. The Gencards database identified 1760 targets, with 1266 remaining after filtering out genes with a Relevance Score below 0.26. The OMIM database yielded 1351 targets. After integrating the two databases for analysis, we identified 1351 key disease-regulating targets. The GO enrichment and KEGG analysis results are shown in Figure 4, which shows the top 10 enriched molecular functions including cytokine receptor binding, receptor ligand activity, signaling receptor activator activity, cytokine activity, growth factor activity, etc. In Figure 4b, the top 10 enriched cellular components are the external side of plasma membrane, vesicle lumen, cytoplasmic vesicle lumen, secretory granule lumen, membrane raft, membrane microdomain, etc. Figure 4c indicates the top 10 biological process including response to lipopolysaccharide, response to molecule of bacterial origin, cytokine-mediated signaling pathway, the regulation of inflammatory response, response to oxidative stress, response to xenobiotic stimulus, etc. Figure 4d displays a summary chart of BP, CC, and MF from GO analysis.

### 3.6. Prediction of Overlapping Targets Between PFOS/PS and Liver Injury

**Figure 5:**
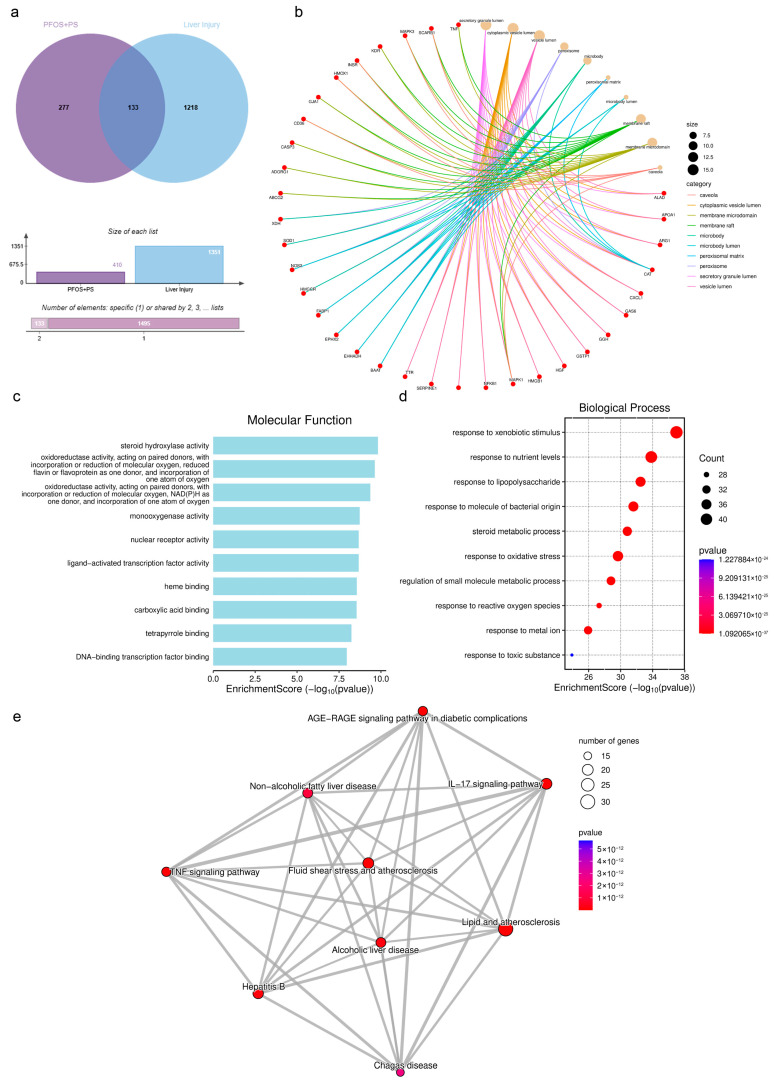
Overlapping predictions between PFOS/PS and liver injury. (**a**): Weisheng Xin online website analysis between PFOS and PS overlapping genes and liver injury; (**b**): cellular component prediction; (**c**): molecular function prediction; (**d**): biological process prediction; (**e**): GO enriched pathway analysis between PFOS and PS overlapping genes and liver injury.

As shown in Figure 5a, by cross-analyzing the combined final targets of PFOS and PS with the obtained disease target prediction data, the Venn diagram revealed 133 overlapping genes between PFOS/PS and liver injury, including *TNF*, *IL6*, *IL1B*, *CASP8*, *BCL2*, *MAPK1*, *CASP3*, *RIPK1*, *BAX*, *MLKL*, *P53*, *CAT*, *GPX4*, *NFKB*, etc. Figure 5b shows the top 10 cellular components of overlapping targets between PFOS/PS and liver injury including the secretory granule lumen, cytoplasmic vesicle lumen, vesicle lumen, peroxisome, microbody, peroxisomal matrix, etc. Figure 5c shows the top 10 molecular functions including steroid hydroxylase activity, oxidoreductase activity, monooxygenase activity, nuclear receptor activity, ligand-activated transcription factor activity, heme binding, etc. Figure 5d shows the differentially expressed genes during PFOS/PS exposure and liver injury, which are enriched in response to xenobiotic stimulus, nutrient levels, lipopolysaccharides, the molecule of bacterial origin, steroid metabolic processes, etc. Figure 5e shows the differentially expressed genes enriched in lipid and atherosclerosis, the *IL-17* signaling pathway, fluid shear stress and atherosclerosis, the *AGE-RAGE* signaling pathway in diabetic complications, the *TNF* signaling pathway, etc.

### 3.7. Genetic Network Analysis of Toxic Components

**Figure 6:**
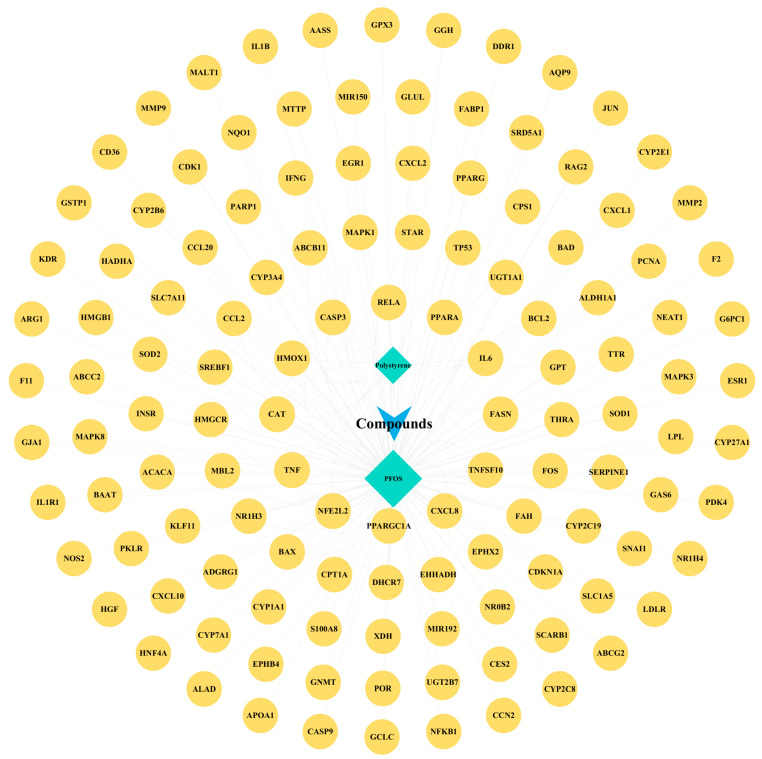
Genetic network analysis of toxic components.

We exported the analysis results from Cytoscape (version 3.9.1), where network nodes represent PFOS, PS, and disease targets. In Figure 6, the maximum degree value in the network indicates stronger correlations between toxins and targets. Green diamonds represent PFOS and PS, with related genes marked by yellow circles. Overlapping genes, *PPARG*, *TNF*, *CAT*, *HMOX1*, *CASP3*, *PPARA*, *IL6*, *CXCL8*, etc., were primarily enriched in apoptosis, necrosis, ferroptosis, inflammatory response, and oxidative stress signaling pathways.

### 3.8. Screening and Prediction of Interactions Between Core Regulatory Proteins

**Figure 7:**
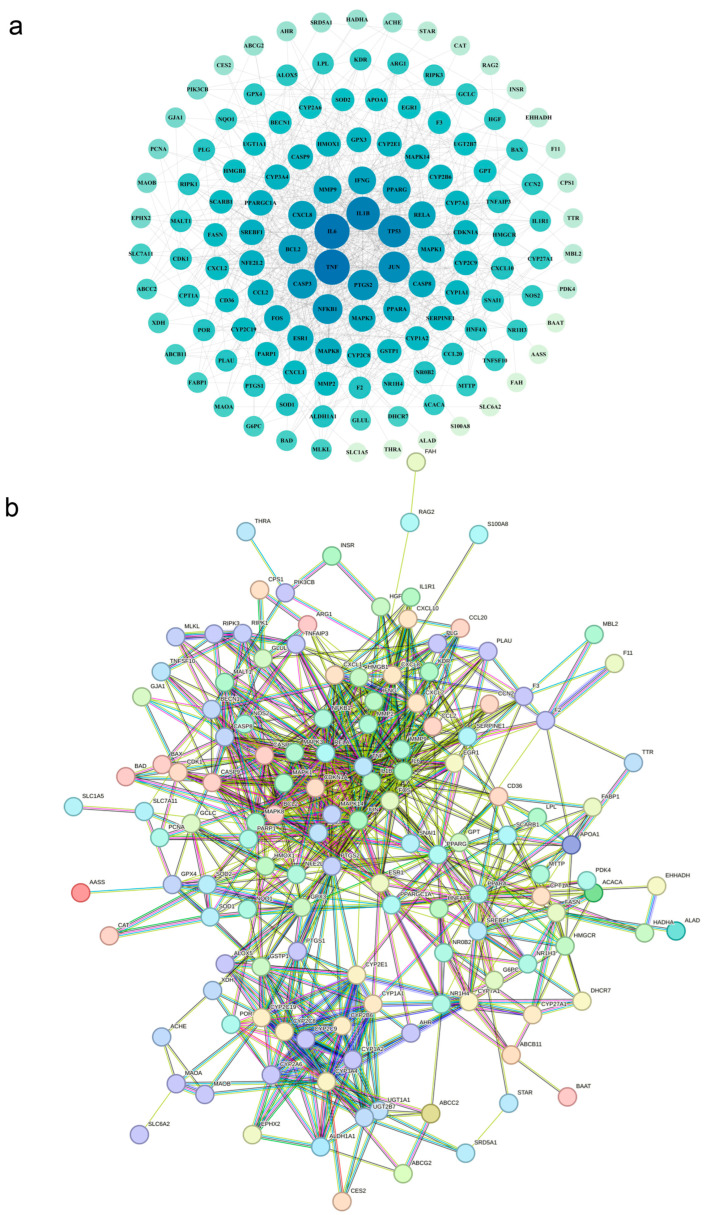
Interactions between core regulatory proteins. (**a**): Common core proteins of PFOS and PS exposure using Cytoscape (version 3.9.1) analysis. (**b**): Combined target proteins of PFOS and PS using STRING database.

As shown in Figure 7a, using Cytoscape 3.9.1 to construct interaction networks, the node sizes corresponding to PFOS and PS targets reflect their biological significance. Larger overlapping protein targets are positioned closer to the network center, with colors progressively approaching dark blue. Higher degree values for core targets indicate greater interaction potential among them. The analysis results revealed that the common core proteins of PFOS and PS exposure include *TNF*, *IL6*, *IL1B*, *TP53*, *JUN,* and *PTGS2*-proteins associated with inflammation and cell death. Additionally, proteins such as *NFKB1*, *CASP3*, *BCL2*, *CXCL8*, *MMP9*, *IFNG*, *PPARG*, *RELA*, *MAPK3*, *CASP8*, and *PPARA*, which regulate apoptosis and lipid metabolism pathways, also play critical regulatory roles in this exposure process.

In addition, as shown in Figure 7b, the STRING database was used to analyze the combined target proteins of PFOS and PS toxins; these results indicate that *APOA1*, *BAD*, *BAX*, *BCL2*, *CASP3*, *CCL2*, *ESR1*, *FOS*, *IL1B*, *NFKB1*, *PPARA*, *PPARG*, etc., are involved in lipid metabolism, apoptosis, and inflammatory pathways and play key roles in the exposure process.

### 3.9. Prediction of Core Protein Interaction Potential for PFOS and PS

**Figure 8:**
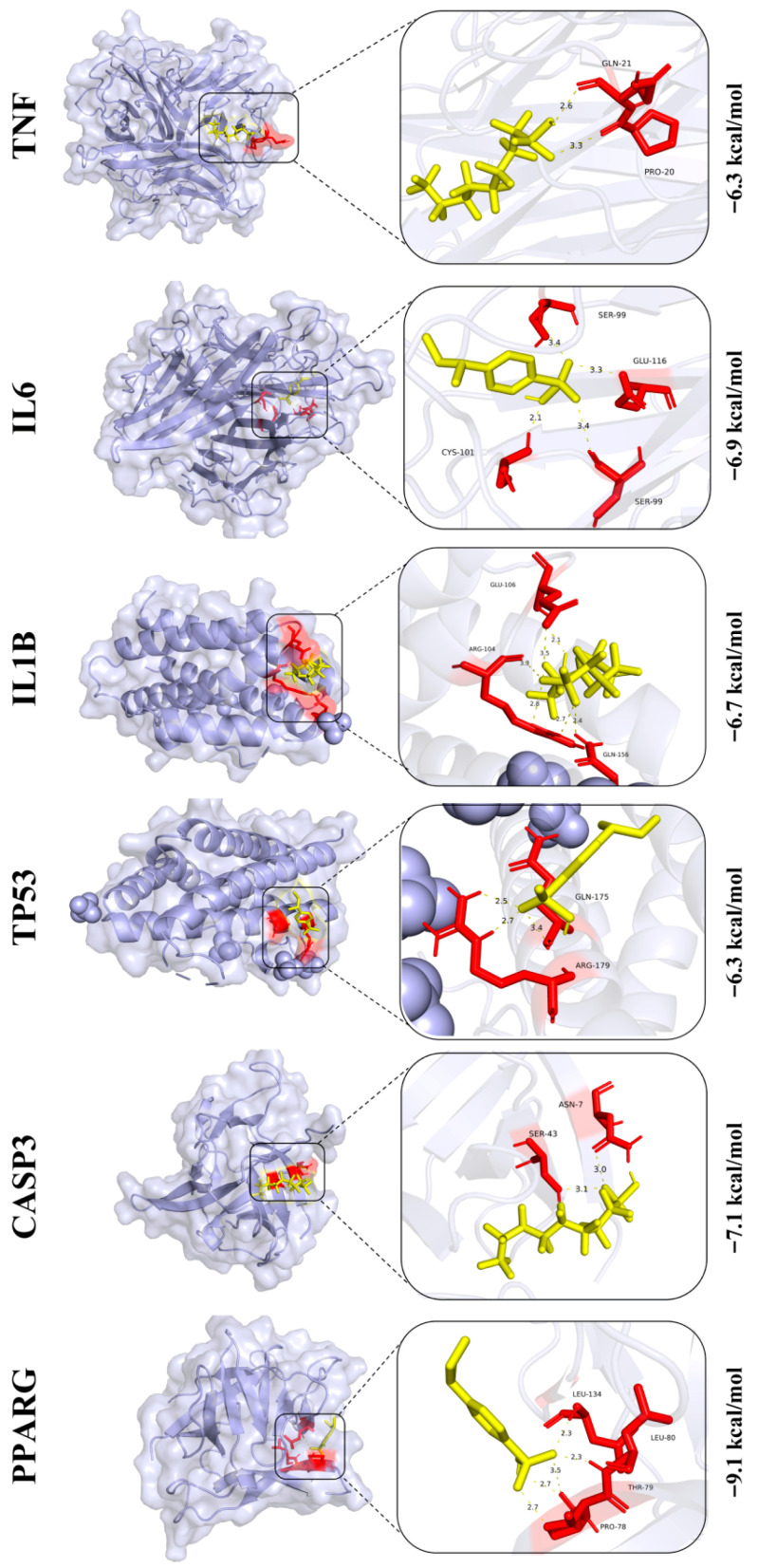
Interactions/binding sites between PFOS and core target proteins. Amino acid residues are shown in red and PFOS in yellow.

**Figure 9:**
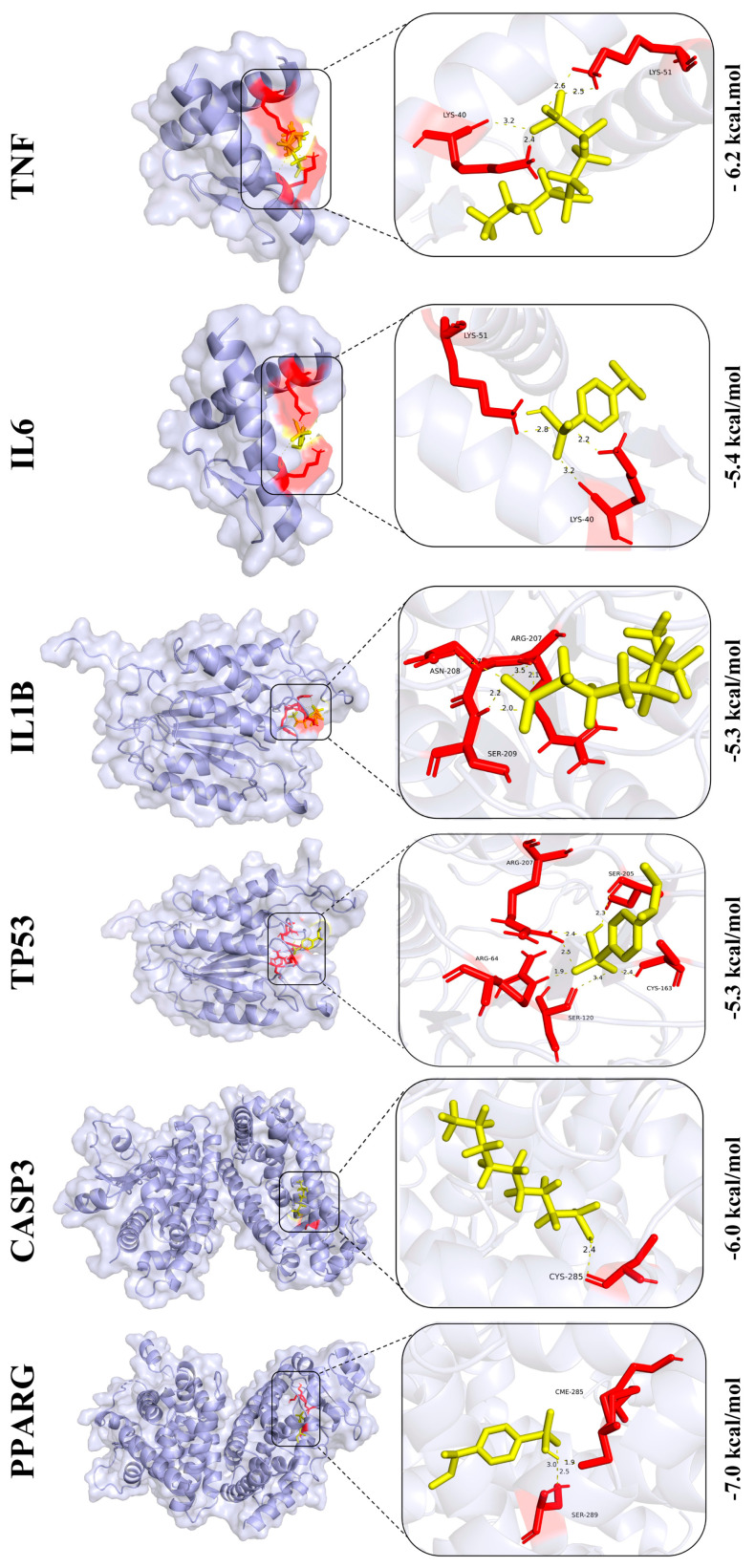
Interactions/binding sites between PS and core target proteins. Amino acid residues are shown in red and PS in yellow.

In Figure 8, the binding energies of PFOS with core proteins *TNF*, *IL6*, *IL1B*, *TP53*, *CASP3*, and *PPARG* are −6.3 kcal/mol, −6.9 kcal/mol, −6.7 kcal/mol, −6.3 kcal/mol, −7.1 kcal/mol, and −9.1 kcal/mol, respectively. As shown in Figure 9, the binding energies of PS and these core proteins are −6.2 kcal/mol, −5.4 kcal/mol, −5.3 kcal/mol, −5.3 kcal/mol, −6.0 kcal/mol, and −7.0 kcal/mol, respectively. Generally, binding energies with negative values indicate stronger interaction potential, where higher absolute values correspond to a greater interaction likelihood. In addition, the predicted binding sites are magnified and displayed in the black box on the right side of the images and detailed binding information are collected in Supplementary Materials.

## 4. Discussion

PFOS is a man-made POP and its stable C-F bonds (with a bond energy of 486 kJ/mol) endow it with exceptional stability, remaining undecomposed even after boiling in concentrated sulfuric acid for one hour. In natural environments, it has a half-life of decades [23]. Due to its hydrophobic, oleophobic, high-temperature-resistant, and surfactant properties, PFOS is widely used in applications such as textile water-repellent finishing agents, firefighting foams, food packaging coatings, and electronic chemicals. Landfill leachate is a primary terrestrial source, while agricultural land irrigated with wastewater serves as a secondary enrichment area. As a persistent organic pollutant, PFOS exhibits liver toxicity, gut toxicity, reproductive system toxicity, neurotoxicity, and endocrine-disrupting effects [24,25,26]. Studies have shown that PFOS causes liver aging damage through the mt-DNA-mediated *NLRP3* signaling pathway [27]. PFOS exposure increases oxidative stress in alligator snapping turtles, which leads to endothelial dysfunction in *ApoE*^−/−^ mice, promoting atherosclerosis formation [28]; it alters lung inflammation and barrier integrity in juvenile mice [29]; and it may promote the occurrence and development of inflammatory bowel disease (IBD) by interfering with key proteins and signaling pathways, highlighting its potential health risks [30]. Studies indicate that PFOS-induced circulating inflammation promotes metabolic remodeling and contractile dysfunction in cardiomyocytes; it also induces p53-mediated apoptosis in splenocytes and thymocytes in C57BL/6 mice [31]. In our previous network toxicology results, PFOS toxicity primarily manifested as genetic toxicity, eye irritation, respiratory system effects, drug-induced nephrotoxicity, and hepatotoxicity. Based on the experimental results in this study, PFOS exposure causes significant liver damage. In the liver, its toxicity mechanisms may include disrupting liver metabolic function—PFOS can inhibit the activity of certain enzymes in the liver, including but not limited to some cytochrome P450 enzymes such as *CYP2C8* and *CYP2C9*. Similarly, PS can inhibit the activity of some CYP450 enzymes, such as *CYP1A2*, *CYP2C19*, *CYP2B6*, and *CYP2C8*, thereby affecting the liver’s metabolic and detoxification functions for toxic substances, inducing oxidative stress. During its metabolism in the body, PFOS generates a large amount of reactive oxygen species (ROS), exceeding the liver’s antioxidant capacity, leading to oxidative stress, which triggers lipid peroxidation, protein oxidation, and DNA damage, activating inflammatory signaling pathways. PFOS can activate inflammatory signaling pathways in the liver, such as the nuclear factor-kappa B (*NF-κB*) pathway, promoting the release of inflammatory factors and triggering chronic inflammatory responses. Based on the network toxicology analysis and experimental results in this study, it is speculated that PFOS exposure can lead to a decrease in the activity and content of antioxidant enzymes, the excessive accumulation of ROS, and consequently cause oxidative damage to the liver.

PS as a primary component of microplastics and is closely linked to human production and life activities in its environmental distribution. Micron-sized PS particles generated from plastic degradation are widely present in water, soil, and the atmosphere. Freshwater systems such as rivers and lakes have become major accumulation zones due to plastic waste input [32]. In marine environments, PS spreads through ocean currents and has been detected in both coastal and deep-sea sediments, where it readily adsorbs heavy metals and organic pollutants to form composite pollution carriers. Additionally, PS can enter remote areas via atmospheric deposition, and its traces have even been found in high-altitude glaciers and polar environments, demonstrating its global cycling characteristics. In terrestrial ecosystems, agricultural land has become a significant source of PS due to plastic film usage and wastewater irrigation, affecting soil microbial community structure and plant growth. Studies have shown that airborne polystyrene nanoplastic exposure leads to heart failure through ECM–receptor interactions and the *PI3K/AKT/BCL-2* pathway [33]; another study indicates that PS microplastics accumulate in the intestines, causing enteritis and posing serious health risks [34]. PS microplastics induce mitochondrial homeostasis imbalance, activate mitochondrial apoptosis pathways, and lead to redox imbalance and increased lipid peroxide accumulation in the liver, further stimulating ferroptosis [35,36]. As an emerging environmental pollutant, the toxicity mechanisms of microplastics primarily include the following aspects: Firstly, as previous studies have shown, polystyrene (PS) microplastics gradually accumulate in human tissues. Through mechanical friction, they cause physical damage to the liver and may inhibit the activity of key liver enzymes [37,38,39]. Secondly, the surface of PS microplastics exhibits chemical adsorption properties, enabling them to trap toxic substances like heavy metals and organic pollutants. This creates composite pollutants that significantly enhance their toxicity to organs. These pollutants may also induce reactive oxygen species (ROS) generation within organs, triggering oxidative stress responses that ultimately lead to cellular damage or even death [40,41]. Additionally, PS microplastics can disrupt the endocrine system, affecting hormone synthesis and metabolic processes [42,43]. Based on network toxicology analysis results, PS exhibits drug-induced neurotoxicity, carcinogenicity, respiratory system damage, and hepatotoxicity. In this study, PFOS and PS reduced the activity and content of antioxidant enzymes, leading to a decreased antioxidant capacity in tissues and the exacerbation of liver oxidative stress, ultimately resulting in histological damage to the liver. However, its effects on other tissues remain to be investigated.

PFOS and PS, as two typical environmental pollutants, are widely present in the natural environment. With the rapid development of industrialization and urbanization, the concentrations of these two pollutants in the environment have gradually increased, and they enter the human body through pathways such as the food chain and drinking water, posing a serious threat to liver health [44]. In recent years, research has found that combined exposure to PFOS and PS may exert synergistic toxic effects on the liver, exacerbating liver damage and leading to liver diseases [45]. Notably, PFOS and PS, as two typical persistent pollutants in the environment, often coexist and form composite pollution. Additionally, these two may physically adsorb or chemically combine to form composite pollutants, such as carrier–pollutant complexes. Studies have shown that the adsorption capacity of PS particles for PFOS can reach 50–200 times compared with the background concentration in water [46]. In addition, PS microplastics generated from the degradation of agricultural plastic films, along with PFOS in irrigation water, enter the soil and may affect microbial community structures. Both PFOS and PS can produce synergistic toxic effects, including the exacerbation of oxidative stress and induce the production of ROS in the liver [45]. During combined exposure, the production of ROS may significantly increase, exceeding the liver’s antioxidant capacity and leading to more severe oxidative stress responses. Furthermore, both PFOS and PS can activate inflammatory signaling pathways in the liver, with the release of inflammatory factors more significant, triggering more severe chronic inflammatory responses. Combined exposure to PFOS and PS may also promote hepatocyte apoptosis through multiple pathways, such as activating the expression of apoptosis-related proteins and inhibiting expressions of anti-apoptotic proteins [47]. This may damage various liver functions, including impaired detoxification function, such as inhibiting the activity of certain enzymes in the liver, affecting the liver’s metabolic and detoxification functions for toxic substances, leading to the accumulation of toxic substances in the body; and impaired synthetic function, since the liver is a site for the synthesis of many important substances in the body, such as proteins and lipids [48]. Combined exposure to PFOS and PS may interfere with the liver’s synthetic function, leading to a reduction in the synthesis of related substances and affecting the body’s normal physiological functions, including impaired secretion function: the liver participates in the digestion and absorption of fats through bile secretion. In this study, we found that, compared with the PFOS-alone exposure group, combined exposure to PFOS and PS significantly exacerbated liver damage, but tissues such as kidney and intestinal also showed varying degrees of damage and reduced antioxidant capacity.

In this study, combined exposure to PFOS and PS induced hepatic balloon degeneration. Compared with the PFOS-only exposure group, combined exposure caused more severe tissue damage, suggesting a potential synergistic effect between the two substances. Additionally, liver antioxidant enzyme T-AOC and CAT activity showed a significant reduction in the combined exposure group compared with the control group and PFOS/PS single exposure groups, while MDA levels exhibited the opposite trend. These findings further validate our hypothesis regarding their synergistic interaction. In conclusion, the unique surface adsorption property of PS in combined PFOS/PS exposure may enhance PFOS retention in the body, thereby intensifying toxic effects.

## 5. Conclusions

In conclusion, when PFOS and PS are co-exposed, PS may exacerbate PFOS-induced antioxidant capacity decline and oxidative stress through its surface-specific adsorption effects, leading to significant liver damage and the partial impairment of kidney and intestinal functions. These findings align with network toxicology research, offering new perspectives and research directions for toxicology while establishing theoretical foundations for environmental monitoring.

## Data Availability

The data that support the findings of this study are available on request from the corresponding author.

## Supplementary Materials

The following supporting information can be downloaded at: https://www.mdpi.com/article/10.3390/biology14121714/s1, Table S1: Relative information of binding box during docking process.
