# Supplementary material for: Multi-Organ Toxicity of Combined PFOS/PS Exposure and Its Application in Network Toxicology
Source: Biology (Basel). 2025 Nov 30;14(12):1714. doi: 10.3390/biology14121714 (PMC12730987; doi:10.3390/biology14121714)
Supplement: Supplementary file 1 [file biology-14-01714-s001.zip › biology-3979019-supplementary.pdf]

Table S1. Relative information of binding box during docking process.

	**CASP3**	**IL1B**	**IL6**	**PPARG**	**TNF**	**TP53**
center_x	26.71	19.495	2.599	-20.153	20.083	52.103
center_y	22.591	2.994	-20.016	0.953	49.892	-12.611
center_z	37.128	73.515	8.749	-25.552	39.738	45.156
size_x	56	40	50	66	62	36
size_y	46	36	50	66	56	40
size_z	62	36	40	68	60	12
energy_range	5	5	5	5	5	5
num_modes	20	20	20	20	20	20
